# Supplementary material for: Allosteric modulation of peroxisomal membrane protein recognition by farnesylation of the peroxisomal import receptor PEX19
Source: Nat Commun. 2017 Mar 10;8:14635. doi: 10.1038/ncomms14635 (PMC5353646; doi:10.1038/ncomms14635)
Supplement: Supplementary Information — Supplementary figures, supplementary tables and supplementary references. [file ncomms14635-s1.pdf]

Supplementary Figure 1

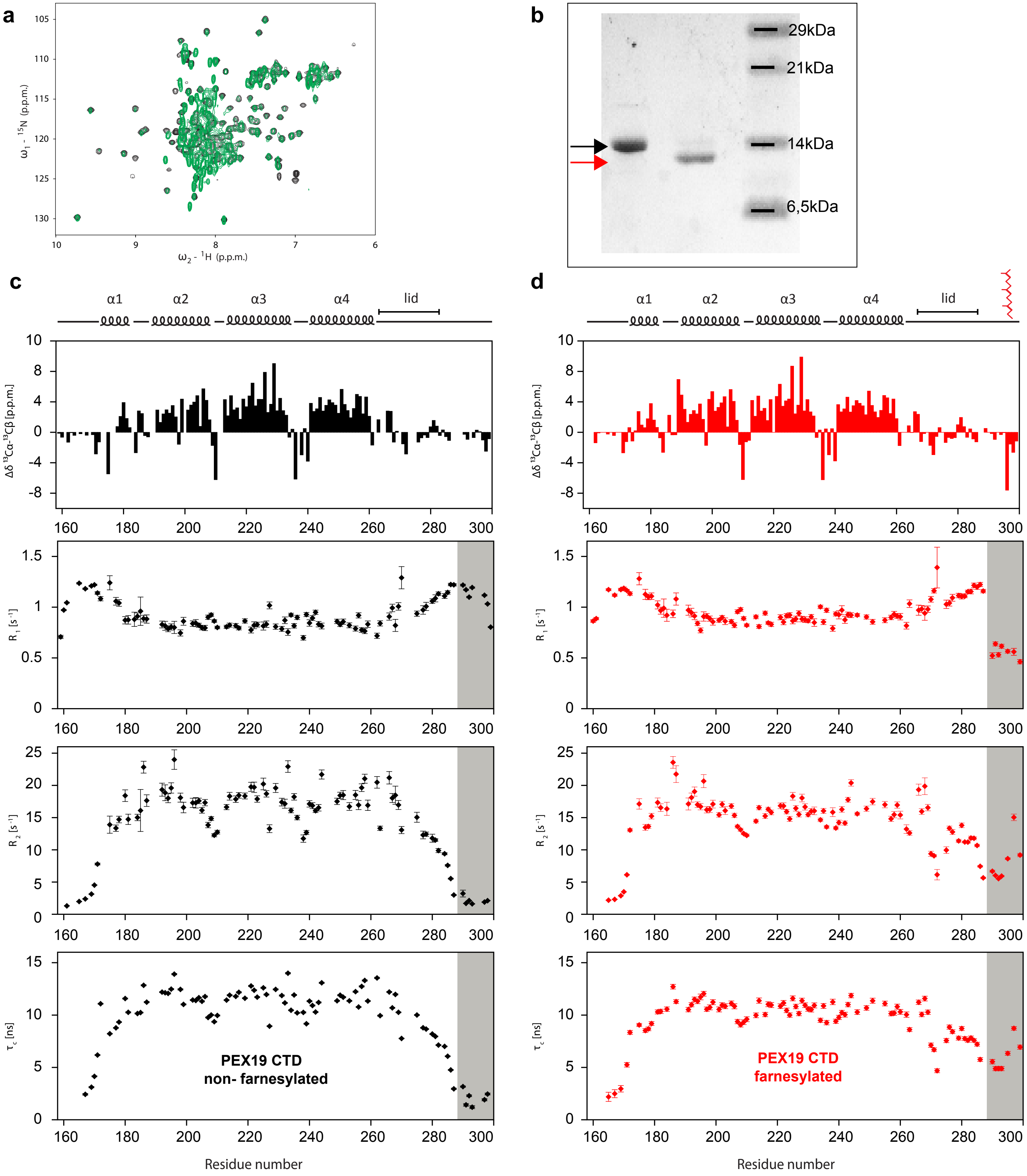

Supplementary Figure 1: Structural analysis of PEX19 protein and farnesylation. (**a**) Overlay of the ^{1}H,^{15}N HSQC NMR spectra of full-length PEX19 (residues 1-299; green) with the C-terminal domain (CTD, residues 161-299; black). Signals from the disordered N-terminus cluster in the center of the spectrum. The chemical shifts for the resolved resonances of the CTD correlate well between the two constructs indicating that there are no strong interactions between the N- and C-terminal regions. (**b**) The farnesylated PEX19 CTD (red arrow) migrates faster on SDS-PAGE than the non-farnesylated (black arrow). (**c**,**d**) NMR analysis of PEX19 CTD farnesylation. Secondary chemical shifts and ^{15}N relaxation data of the PEX19 CTD without (**c**) and with farnesylation (**d**). Error bars represent fitting deviations. The ^{13}C secondary chemical shifts (upper panel) indicate four α-helices for the non-farnesylated and the farnesylated protein. ^{15}N *R1* and *R2* relaxation rates and local tumbling correlation times τ_c derived from these data indicate large differences induced by farnesylation for the C-terminal residues (highlighted with grey boxes). Secondary structure elements, derived from the NMR structure of the farnesylated PEX19 and from the crystal structure of the non-farnesylated PEX19, are indicated on top together with the farnesylation site. Structures and $^{13}C^{\alpha/\beta}$ chemical shifts for both forms of PEX19 agree that the α-helical regions are not altered by farnesylation.

Supplementary Figure 2

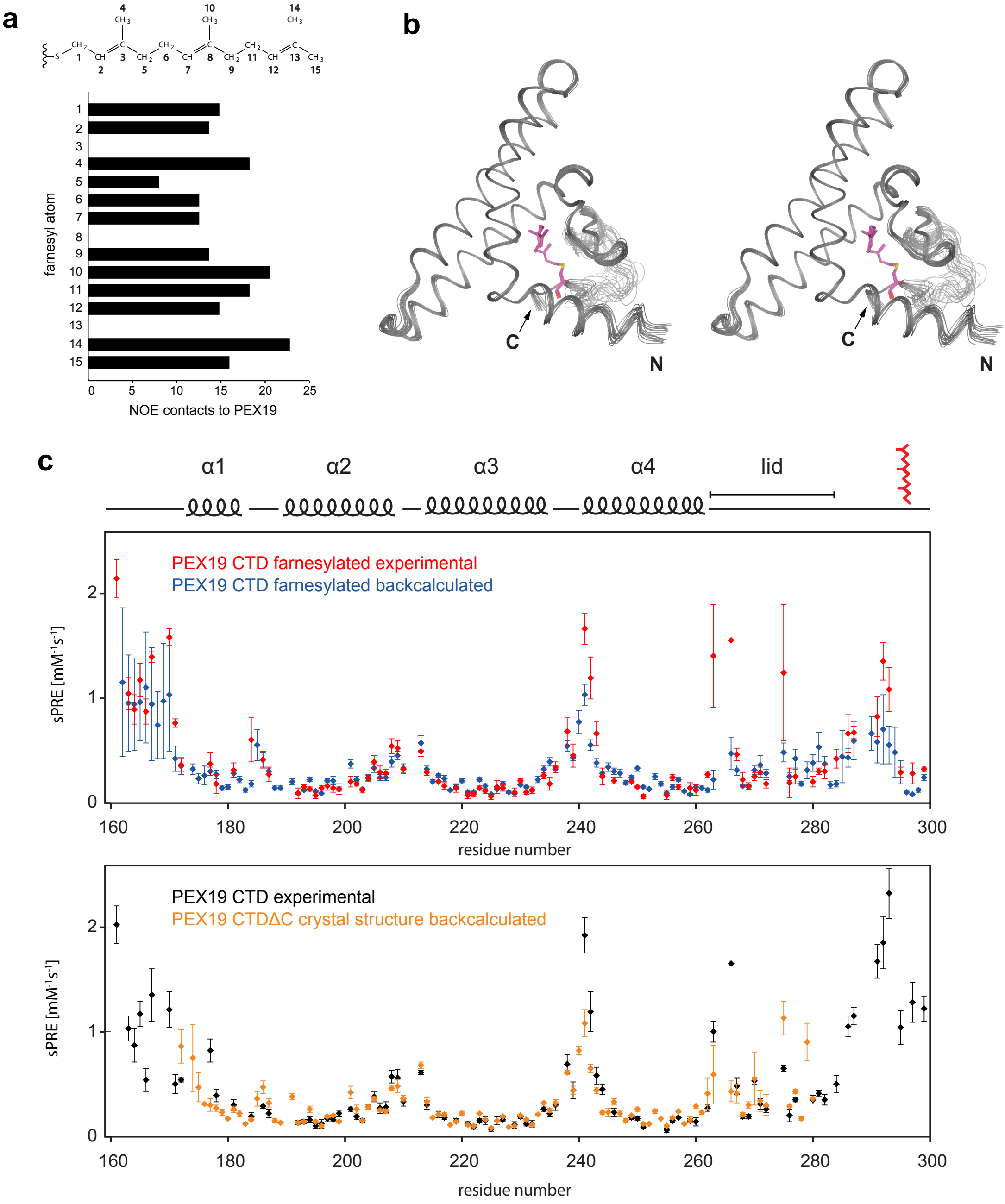

Supplementary Figure 2 Structure ensemble of farnesylated PEX19 CTD and validation. Initial isotope-filtered NOE experiments indicated that the protein-farnesyl interaction involves mainly methyl groups of aliphatic side chains in the PEX19 CTD. Since the CTD contains 17 leucine, 6 isoleucine, and 9 methionine residues, several signals from the methyl groups of these amino acids could not be unambiguously assigned due to signal overlap. To resolve ambiguities for key residues that are in contact with the farnesyl group an optimized isotope labeling strategy was used combining amino acid-selective labeling, specific methyl labeling [45], and reverse labeling of phenylalanines [46]. $^{1}H,^{13}C$ correlations of the farnesyl group were obtained at natural abundance of ^{13}C with uniformly deuterated protein. Using this strategy, we achieved complete resonance assignments for PEX19 CTD and the farnesyl group. (**a**) NOESY spectra of selectively labeled farnesylated PEX19 CTD samples unambiguously identify the contacts between farnesyl protons and PEX19 CTD. The graph shows the numerous NOEs for each farnesyl proton to PEX19 CTD that define the binding site and farnesyl conformation. Farnesyl atom numbers are indicated in the chemical formula above. (**b**) Stereoview of the ensemble of the 20 lowest energy NMR structures of farnesylated PEX19 CTD. The farnesyl moiety is shown in magenta. (**c**) Comparison of back-calculated and experimental solvent PREs. Experimental solvent PRE of backbone amide protons of farnesylated PEX19 CTD (upper panel, red) and non-farnesylated PEX19 CTDΔC (lower panel, black) were compared to values back-calculated from the ensemble of NMR structures of farnesylated PEX19 CTD (upper panel, blue) and the crystal structure of PEX19 CTDΔC (lower panel, orange). Error bars of experimental and the back-calculated data represent fitting errors and PRE s.d. (for the NMR ensemble or crystal monomers), respectively. Secondary structure elements are indicated on top. Values for the core region including helices α2-α4 are in excellent agreement. For flexible regions, experimental values are higher than the back-calculated due to chemical exchange between amide protons in these parts with water molecules, which experience a large relaxation enhancement because of transient binding to Gd(DTPA-BMA). Deviations between experimental values for non-farnesylated PEX19 CTDΔC and the back-calculated rates for the crystal structure are found in helix α1 and the residues in the C-terminal residues of the crystal structure from residue 278-281 indicating that the conformation in solution is not entirely reflected by the crystal structure.

Supplementary Figure 3

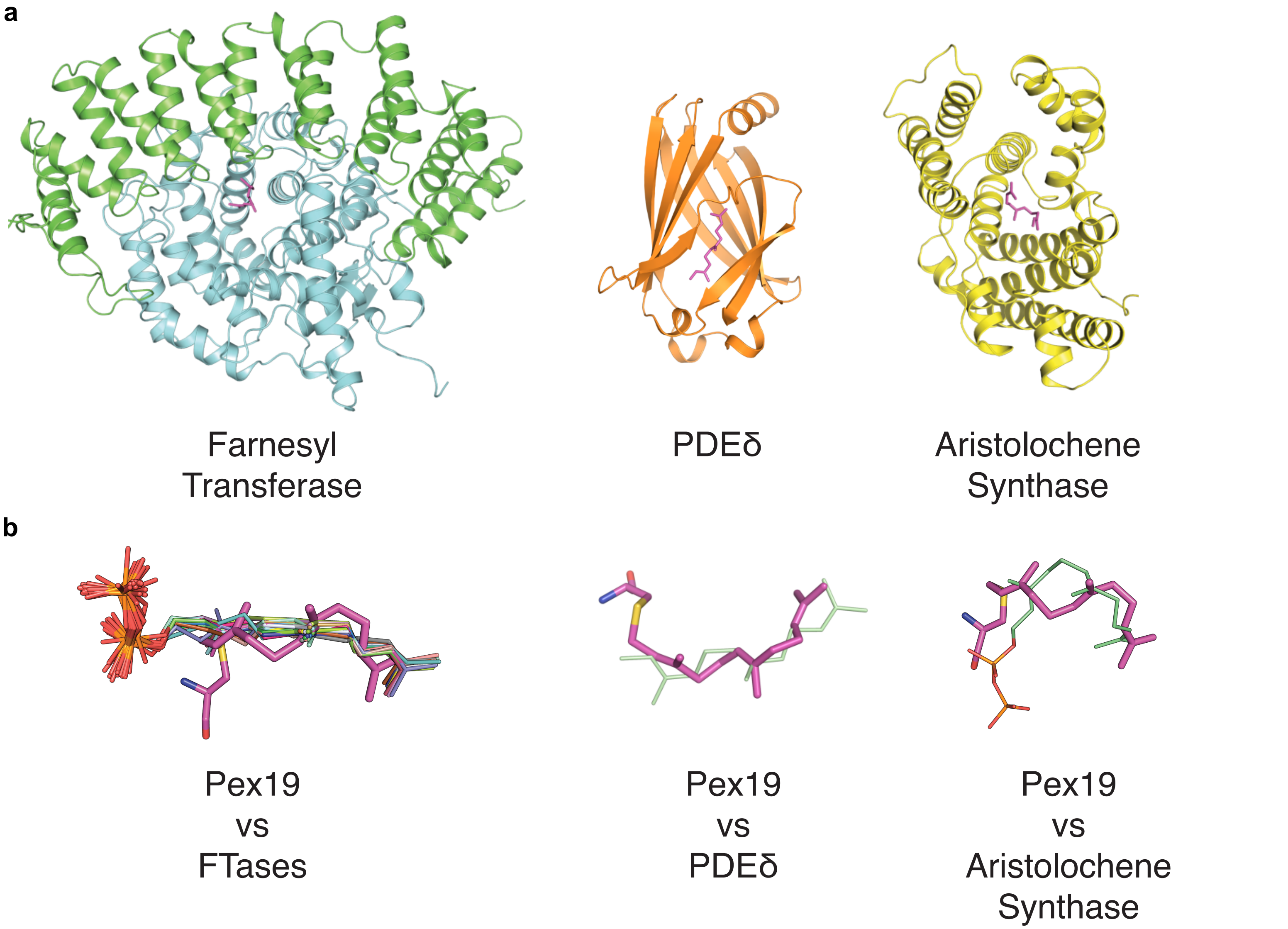

Crystal structures
1JCQ 1JCR 1KZO 1LD8
1LD7 1MZC 1O5M 1S63
1SA4 1SA5 2BED 2F0Y
2R2L 2ZIR 2ZIS 3DPY
3E30 3E32 3E33 3E34
3KSQ 3Q7A 3Q7F

Crystal structure
3T5G

Crystal structure
3BNX

Supplementary Figure 3: Farnesyl conformations adapted in different proteins. (**a**) Overall fold of farnesyl transferase, solubilizing factor PDEδ and aristolochene synthase accommodating in hydrophobic pockets the farnesyl moiety (shown with magenta sticks). (**b**) Structural comparison of the PEX19 farnesyl conformation (thick magenta sticks) with the extended conformations seen in 23 farnesyl transferase structures (left), with the extended farnesyl conformation when bound by PDEδ (middle), and with the conformation seen when bound by aristolochene synthase that shows a similar bent conformation (right). The PDB codes of the crystal structures used for comparing the farnesyl conformation are listed, and those for preparing the cartoon representations are highlighted in red. See text for references.

Supplementary Figure 4

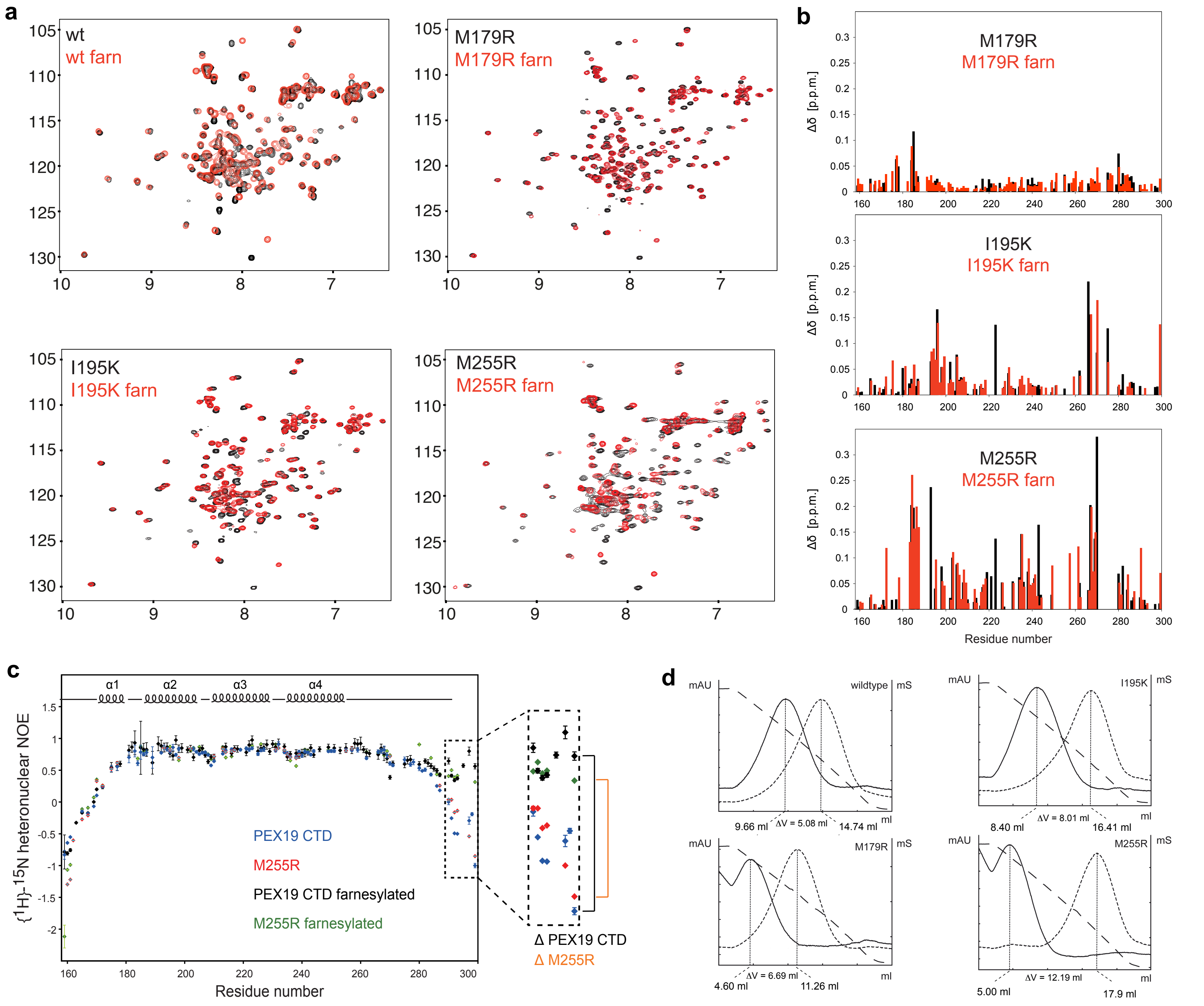

Supplementary Figure 4: NMR and hydrophobicity analysis of PEX19 CTD variants that affect farnesyl recognition. (**a**) Superposition of ^{1}H,^{15}N HSQC spectra of non-farnesylated (black) and farnesylated (red) PEX19 CTD proteins (wild type, M179R, I195K, and M255R mutants). Protein concentration for all mutants was 50 μM and for wild type was 100 μM. (**b**) Chemical shift perturbations plotted for wild type versus residue specific mutations for the non-farnesylated (black) and farnesylated (red) forms. (**c**) {^{1}H}-^{15}N heteronuclear NOE values for PEX19 CTD M255R with and without farnesylation compared to those of the wild type protein. Error bars are based on s.d. of noise in the individual spectra and were calculated using error propagation. While the globular helical region of the protein is highly similar, the farnesylation-induced rigidity of the C-terminal residues seen for the wild type protein is significantly less for PEX19 CTD M255R. (**d**) Hydrophobic interaction chromatography. PEX19 CTD and variants were subjected to hydrophobic interaction chromatography using Butyl Sepharose FF. Bound proteins were eluted with linear decreasing $(NH_4)_2SO_4$ concentrations shown by decreasing conductance (mS, dashed line). Eluted proteins were detected by 280 nm absorption (mAU). Comparison of farnesylated (dotted line) and unmodified proteins (solid line) reveals a shift in the elution volume pointing to an increase of hydrophobicity due to farnesylation. The elution volume for every protein after start of the gradient and the difference between the farnesylated and non-farnesylated proteins (in ml) are indicated below each graph. Mutants affecting farnesyl binding (M179R, I195K, M255R) show increasing shifts in the elution volume.

Supplementary Figure 5

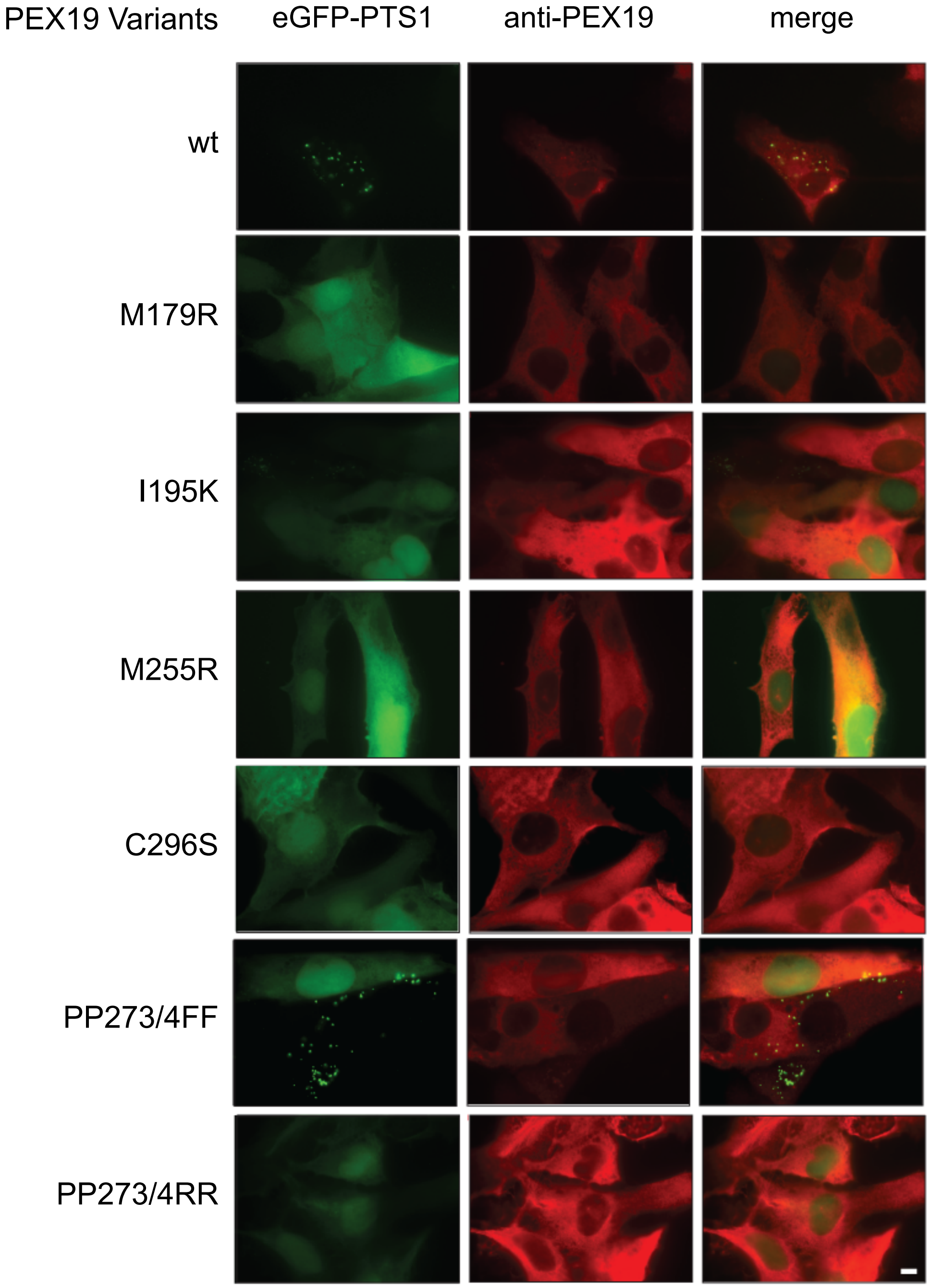

Supplementary Figure 5: Subcellular localization of PEX19 mutants in vivo. Immunufluoresence microscopy images of PEX19 deficient human fibroblasts transfected with bicistronic vectors encoding for the peroxisomal matrix protein eGFP-SKL (green) and different PEX19 variants used in this study. 72 hours after transfection, cells were immuno-labelled with anti PEX19 antibodies (red). A punctate pattern of eGFP-SKL tags import compentent peroxisomes which also associates with a small fraction of PEX19. All cells which were not complemented by expression of PEX19 variants showed a cytosolic staining of PEX19 without association with peroxisomes or other subcellular compartments. Scale bar: 10mm.

Supplementary Figure 6

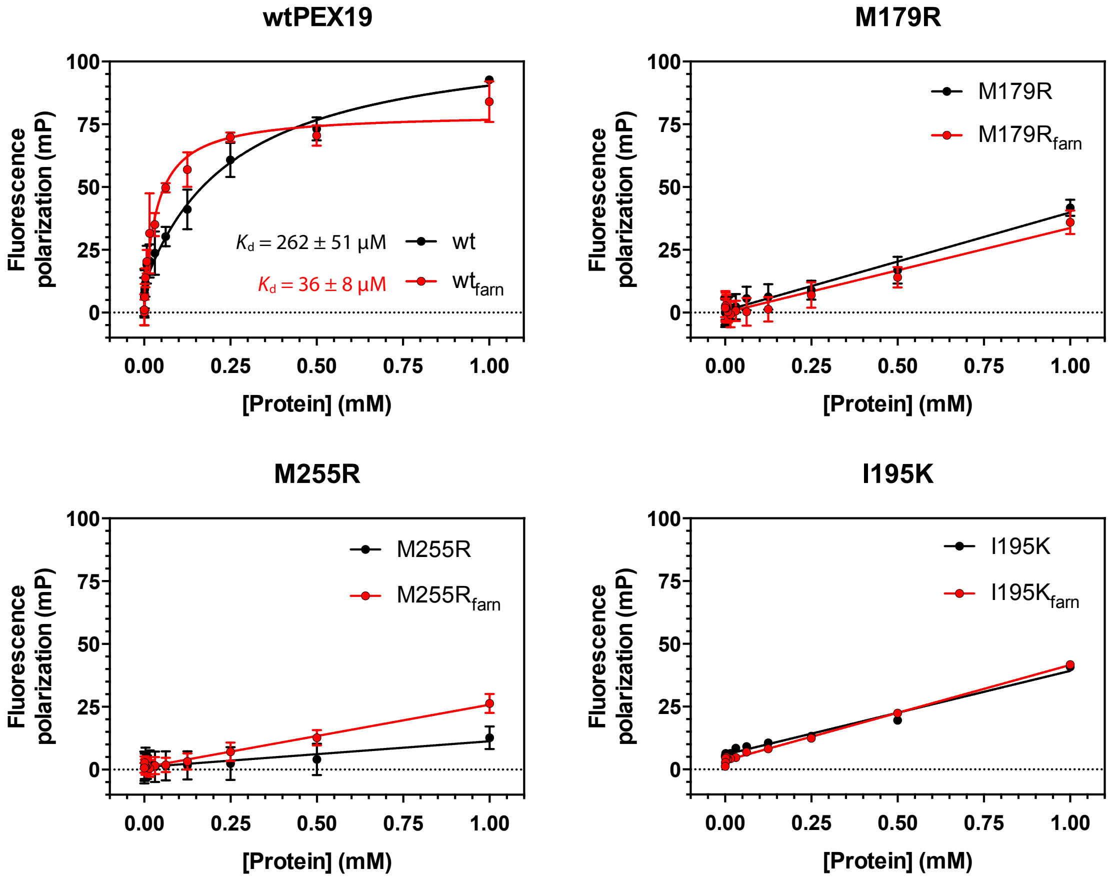

Supplementary Figure 6: ALDP affinity measurement for PEX19 CTD proteins using a fluorescence polarization assay. Polarization plots as a function of protein concentration for the direct titration of PEX19 non-farnesylated (black) and farnesylated (red) forms (wild type and residue spedific mutants) into fluorescein-labeled ALDP peptide. Fitted curves revealed a seven fold increase in affinity for wild type PEX19 CTD upon farnesylation, while all mutants showed impaired binding with or without farnesylation. Error bars indicate standard deviations for triplicate measurements (n = 3).

Supplementary Figure 7

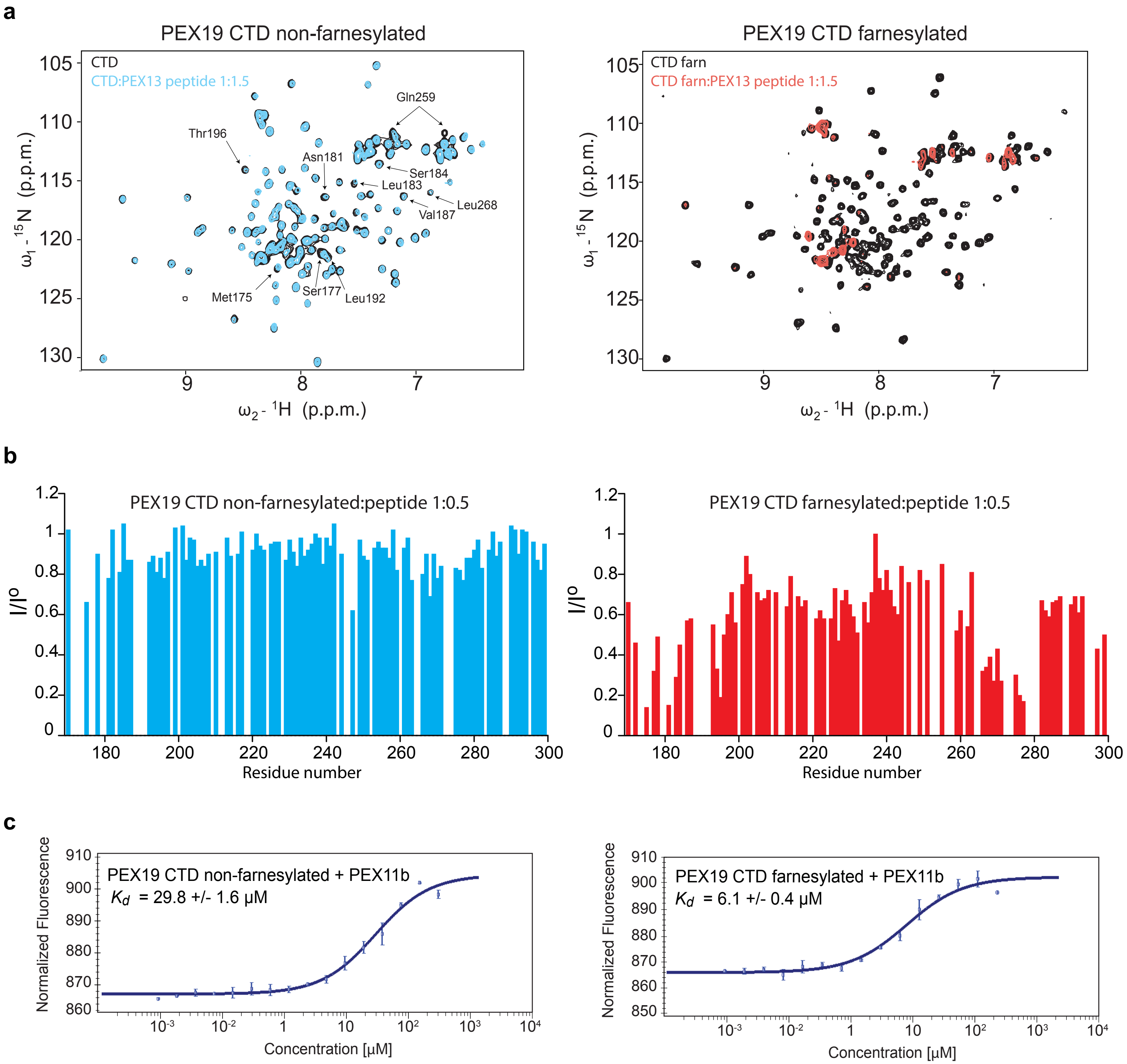

Supplementary Figure 7: Binding of PEX19 CTD to PMP peptides. (**a**) Superposition of NMR spectra of non-farnesylated and farnesylated PEX19 CTD with the PEX13 peptide at 1:1.5 molar ratio. 50 µM of PEX19 CTD was used for all spectra. (**b**) Analysis of the intensity differences upon binding of non-farnesylated (left) and farnesylated (right) PEX19 CTD to the PEX13 peptide (sequence: HFTKVFSAFALVRTIR) at 1:0.5 molar ratio. (**c**) Microscale thermophoresis assays. 100 nM N-terminally fluorescein-labeled peptide PEX11b (sequence: LALKLRLQVLLLARV) was used to monitor interactions with unlabeled non-farnesylated and farnesylated Pex19 CTD using a NanoTemper Monolith NT 0.15T instrument (NanoTemper GmbH, Munich, Germany). Normalized fluorescence values from three separate measurements were used to determine dissociation constant (K_d) values. The error bars represent s.d. of independent measurements.

Supplementary Figure 8

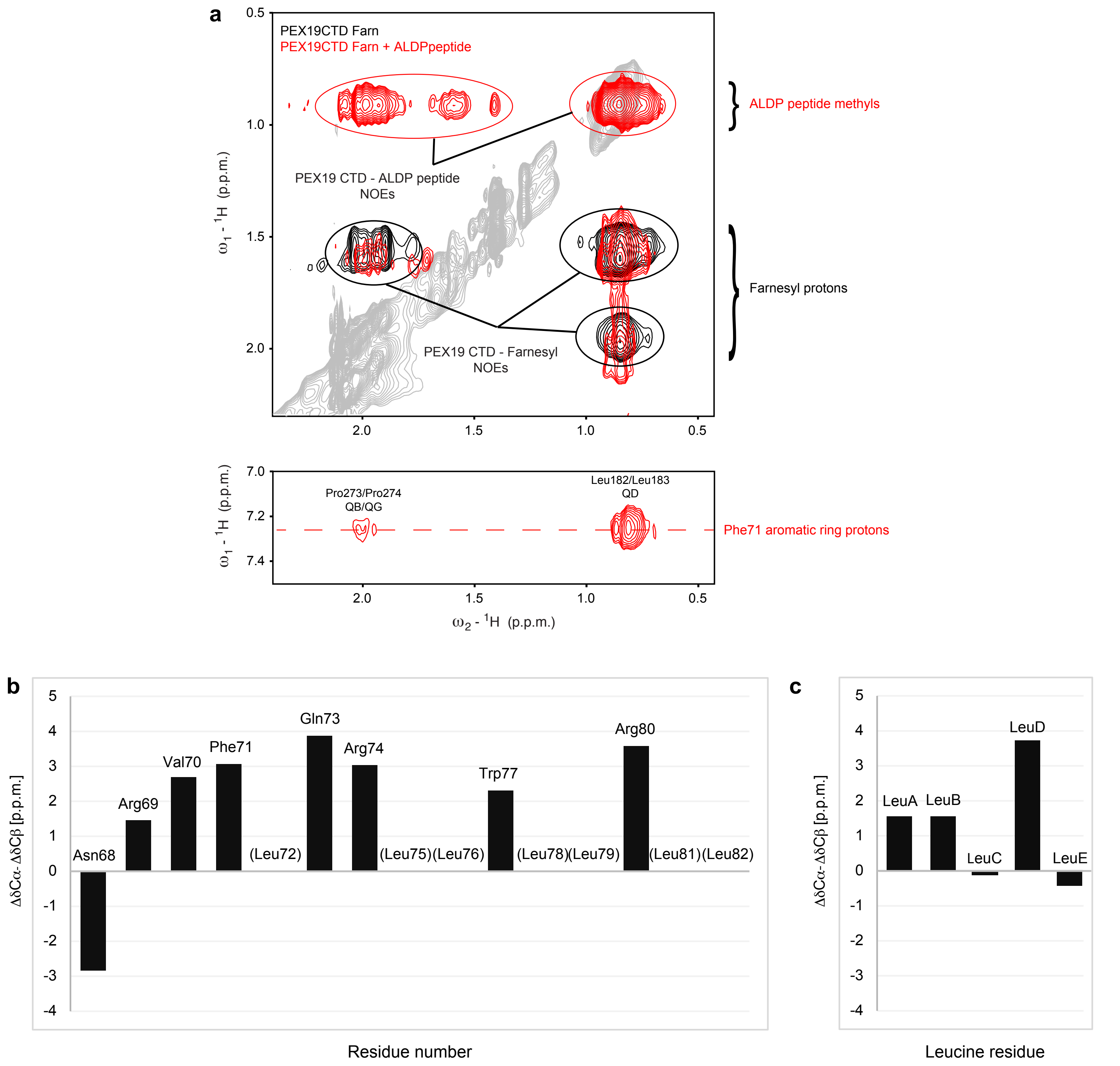

Supplementary Figure 8: Intermolecular NOEs between PEX19 CTD and the ALDP peptide, and secondary structure of the latter. (**a**) ^{1}H,^{1}H plane of a 3D ω_1-filtered, ω_2-edited ^{13}C-NOESY-HSQC experiment of farnesylated PEX19 CTD free (black) and bound to the ALDP peptide (red). Methyl and methylene protons of the unlabeled farnesyl group of the PEX19 CTD show NOEs with protons of the isotope-labeled farnesylated PEX19 CTD (black circles). In the presence of also unlabeled ALDP peptide additional intermolecular methyl-methyl and aliphatic-aromatic NOEs are observed (red circles and below). (**b**) NMR ^{13}C secondary chemical shifts of a C-terminally extended version of the ALDP peptide, which harbors an additional tryptophan (residue 68 to 82) indicate that it adopts a largely helical conformation. (**c**) Due to signal overlap, 5 out of 7 spin systems for leucine residues were assigned. Nevertheless, also the leucines residues adopt a helical conformation.

Supplementary Figure 9

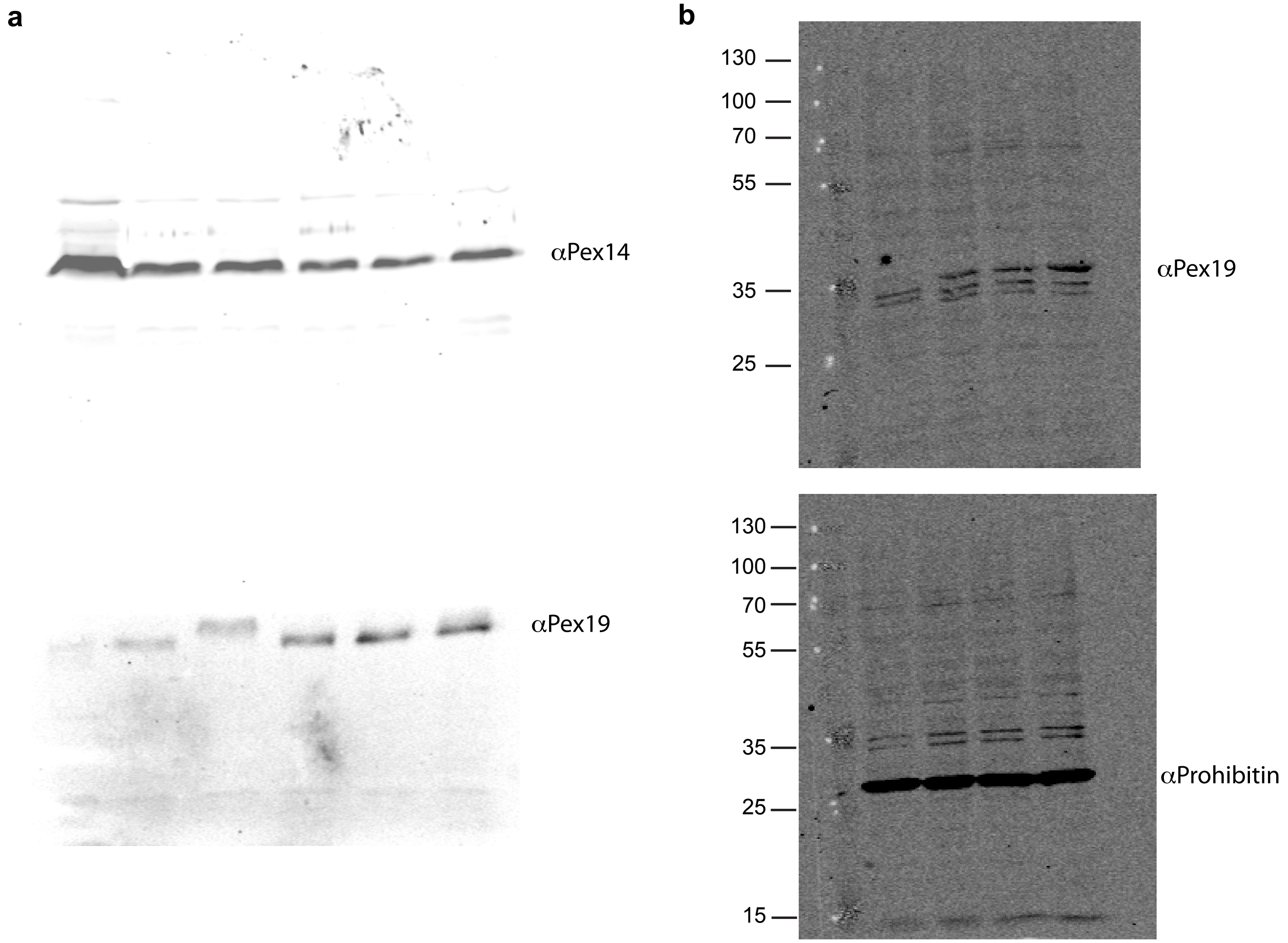

Supplementary Figure 9: Uncropped blots shown in the main figures. (**a**) The uncropped western blots shown in Figure 3d. (**b**) The uncropped western blots shown in Figure 5e.

Supplementary Table 1. Sequences of oligonucleotide primers for PEX19 CTD mutagenesis

Mutation	Forward primer (5' – 3')	Reverse primer (5' – 3')
M179R	CCTCCCCATCATGCAGAGTATTAGGCAGAACCTACT	AGTAGGTTCTGCCTAATACTCTGCATGATGGGGAGG
I195K	TGTGCTGTACCCATCACTGAAGGAGAAGACAGAAAAGTATCC	GGATACTTTTCTGTCTTCTCCTTCAGTGATGGGTACAGCACA
M255R	TTTTGAGATGGTGCTGGATCTTAGGCAGCAGCTACA	TGTAGCTGCTGCCTAAGATCCAGCACCATCTCAAAA
C296S	GTGCAAGTGGTGAACAGAGTCTGATCATGTGATAA	TTATCACATGATCAGACTCTGTTCACCACTTGCAC
P273R	GTCAAAGTTGAGGCCAGGTCTCATCTCTCCAGCCAGCTC	GAGCTGGCTGGAGAGATGAGACCTGGCCTCAACTTTGAC
P273F	GTCAAAGTTGAGGCCAGGGAACATCTCTCCAGCCAGCTC	GAGCTGGCTGGAGAGATGTTCCCTGGCCTCAACTTTGAC
P274R	TCAAAGTTGAGGCCTCTAGGCATCTCTCCAGCCAGCTC	GAGCTGGCTGGAGAGATGCCTAGAGGCCTCAACTTTGA
P274F	GTCAAAGTTGAGGCCGAAAGGCATCTCTCCAGCCAGCTCT	AGAGCTGGCTGGAGAGATGCCTTTCGGCCTCAACTTTGAC
PP 273/4 FF	GGCATCCAGGTCAAAGTTGAGGCCGAAGAACATCTCTCCAGCCAGCTCTTTTGG	CCAAAAGAGCTGGCTGGAGAGATGTTCTTCGGCCTCAACTTTGACCTGGATGCC
PP 273/4 RR	GCATCCAGGTCAAAGTTGAGGCCTCTTCTCATCTCTCCAGCCAGCTCTTTTG	CAAAAGAGCTGGCTGGAGAGATGAGAAGAGGCCTCAACTTTGACCTGGATGC

Supplementary Table 2: Statistical comparison of two best HADDOCK clusters

	HADDOCK statistics	
Parameter	Cluster2	Cluster5
	Best 4 models	Best 4 models
HADDOCK score	-38.8	-33.3
Cluster size	19	7
i-RMSD[a]	0.6 Å	4.15 Å
l-RMSD[b]	3 Å	28 Å
fnat[c]	0.85	0.47
VdW[d]	-23.1 kcal/mol	-20.9 kcal/mol
Elec[e]	-111.5 kcal/mol	-131.9 kcal/mol
Violation[f]	5.2 kcal/mol	11.7 kcal/mol
BSA[g]	752.5 Å^2	716.1 Å^2

[a] interface root mean square deviation
[b] ligand root mean square deviation
[c] fraction of native contacts
[d] Van der Waals contribution to intermolecular energies
[e] electrostatic contribution to intermolecular energies
[f] restrains violation energy
[g] buried surface area

Supplementary References

1. Tugarinov, V., Kanelis, V. & Kay, L. E. Isotope labeling strategies for the study of high-molecular-weight proteins by solution NMR spectroscopy. *Nat. Protoc.* **1**, 749-754 (2006).

2. Vuister, G. W., Kim, S.-J., Wu, C. & Bax, A. 2D and 3D NMR Study of Phenylalanine Residues in Proteins by Reverse Isotopic Labeling. *J. Am. Chem. Soc.* **116**, 9206-9210 (1994).
